# PU-DZMS: Point Cloud Upsampling via Dense Zoom Encoder and Multi-Scale Complementary Regression

**DOI:** 10.3390/jimaging11080270

**Published:** 2025-08-12

**Authors:** Shucong Li, Zhenyu Liu, Tianlei Wang, Zhiheng Zhou

**Affiliations:** 1 School of Information Engineering, Guangdong University of Technology, Guangzhou 510006, China; 2112403038@mail2.gdut.edu.cn; 2 School of Electronic and Information Engineering, Wuyi University, Jiangmen 529020, China; tianlei.wang@aliyun.com; 3 School of Electronic and Information Engineering, South China University of Technology, Guangzhou 510630, China; zhouzh@scut.edu.cn

**Keywords:** point cloud imaging, point cloud upsampling, Dense Zoom Encoder, Multi-Scale Complementary Regression

## Abstract

Point cloud imaging technology usually faces the problem of point cloud sparsity, which leads to a lack of important geometric detail. There are many point cloud upsampling networks that have been designed to solve this problem. However, the existing methods have limitations in local–global relation understanding, leading to contour distortion and many local sparse regions. To this end, PU-DZMS is proposed with two components. (1) the Dense Zoom Encoder (DENZE) is designed to capture local–global features by using ZOOM Blocks with a dense connection. The main module in the ZOOM Block is the Zoom Encoder, which embeds a Transformer mechanism into the down–upsampling process to enhance local–global geometric features. The geometric edge of the point cloud would be clear under the DENZE. (2) The Multi-Scale Complementary Regression (MSCR) module is designed to expand the features and regress a dense point cloud. MSCR obtains the features’ geometric distribution differences across scales to ensure geometric continuity, and it regresses new points by adopting cross-scale residual learning. The local sparse regions of the point cloud would be reduced by the MSCR module. The experimental results on the PU-GAN dataset and the PU-Net dataset show that the proposed method performs well on point cloud upsampling tasks.

## 1. Introduction

Point cloud imaging technology captures the 3D spatial coordinates of objects, enabling fundamental 3D reconstruction and analysis capabilities for diverse applications such as environmental perception for autonomous driving, surveying and modeling, industrial quality inspection, and digital preservation of cultural relics.

However, a point cloud obtained by imaging sensors is usually sparse, which leads to a lack of geometric information regarding the instances [1]. This situation might be caused by the hardware limitations of the imaging equipment. Therefore, point cloud upsampling is an effective method to recover the original point cloud distribution.

There are many studies focusing on point cloud upsampling. They could be categorized into point-based, attention-based, and convolution-based methods. The pioneering point-based methods include the seminal PU-Net [1] framework, later extended by approaches like MPU [2], which introduced multi-step progressive upsampling to enhance detail preservation, particularly for distant representations. Attention-based methods emerged with PUT [3], the first to leverage Transformer architectures for point cloud upsampling. Subsequent refinements like PU-Edgeformer [4] further encoded the query, key, and value tensors derived from a self-attention mechanism. For convolution-based methods, PU-GCN [5] employed graph convolutional networks to extract detailed neighborhood information and generate points in expanded spatial contexts, while PU-GAN [6] incorporated generative adversarial training to achieve superior point distribution uniformity. PUGeo [7] represented another significant advancement by explicitly learning fundamental geometric forms to exploit intricate local surface relationships. Additional methods like PU-Flow [8], Dis-PU [9], and that of Li et al. [10] utilized graph or dense convolution backbones to capture complex feature representations across diverse point structures effectively. PUDM [11] addressed geometric detail loss in sparse inputs by modeling dense point distribution gradients through a dual mapping paradigm, enabling arbitrary-scale point cloud upsampling.

Although the existing approaches have achieved progress in extracting global features and modeling surfaces, they suffer from key limitations in local–global relation understanding. This issue arises due to the following factors. (1) The existing approaches obtain local and global features, respectively, and fail to fuse them adequately. (2) The existing approaches rely on processing data at only one scale and use single-step regression strategies. These limitations would result in contour distortion and many local sparse regions in the generated point cloud.

To this end, PU-DZMS is proposed to achieve the generation of dense and high-quality point clouds. PU-DZMS contains two parts. First, the Dense Zoom Encoder (DENZE) is designed to extract the local–global geometric features of point clouds. The main module in the DENZE is the ZOOM Block, which consists of three dense connection Zoom Encoders. The Zoom Encoder embeds a Transformer mechanism into the process of down–upsampling to enhance the local geometric features. Second, the Multi-Scale Complementary Regression (MSCR) module is designed to expand the encoded features and regress the dense point cloud. In MSCR, expanded features are obtained by referring to the geometric distribution difference regarding point clouds under different scale factors. Cross-scale residual learning is adopted to regress new points from the expanded features. MSCR could reduce the occurrence of local blank spaces effectively. The experimental results on the PU-GAN dataset and the PU-Net dataset show that our model could perform well on point cloud upsampling tasks.

In short, our main contributions are as follows:

• A novel point cloud upsampling network named PU-DZMS is proposed, which could avoid outline distortion and reduce local sparse regions in the dense point cloud.
• The DENZE is designed to learn latent local–global geometric features by employing serial ZOOM Blocks with a dense connection. The ZOOM Block contains Zoom Encoders, which embed a Transformer into the process of down–upsampling to achieve local feature enhancement.
• The MSCR module is designed to expand features and regress dense point clouds. This module combines multi-scale geometric distribution differences and cross-scale residual learning, ensuring the completeness of the dense point cloud.
• PU-DZMS is evaluated on the PU-GAN dataset and the PU-Net dataset. The experimental results show that our model could generate dense and high-quality point clouds.

## 2. Related Work

### 2.1. Point Cloud Upsampling

Many works focus on point cloud upsampling or 3D point cloud reconstruction. These works are usually classified into point-based, attention-based, and convolution-based methods. For point-based methods, PU-Net [1] and 3PU [2] are the pioneer works based on PointNet++ [12] for point cloud upsampling. For attention-based methods, PUT [3] and PU-Edgeformer [4] introduced the Transformer mechanism to capture long-range dependencies for upsampling. For convolution-based methods, PU-GCN [5] adopted a graph convolution network to extract the neighbor information of points, thus learning new points from a new space. Others, like [8,9,10], adopted graph or dense convolution as a backbone to grasp features effectively. PUDM [11] leveraged conditional DDPM with dual mapping and rate priors for upsampling, demonstrating good noise resilience.

However, these works captured local details adequately but had inefficient modeling in a global perspective. Moreover, they focused on single-scale modeling and regression, where geometric discontinuities would bring local sparse regions, reducing the quality of the generated point cloud.

### 2.2. Point Cloud Feature Learning

Point clouds are characterized by disorder and irregularity. To adequately learn the features of point clouds, [13] proposed the initial network named PointNet to extract a point-wise feature by shared-MLP and aggregated all the features into a global representation. Later, based on a hierarchical point set feature learning concept, PointNet++ [12] was proposed, which obtains multi-scale features by introducing a set abstraction layer. In comparison with PointNet, PointNet++ not only focuses on both local and global features but also has an improved generalization ability for the network.

Besides [12,13], some works adopted a CNN architecture to learn the features of point clouds. PointCNN [14] utilized X-Conv to transform raw point clouds into an ordered representation; by this method, a CNN can be employed on raw point clouds to extract features. In [15], the EdgeConv unit was designed to learn the local features of raw point clouds, and the global information can be further extracted through several EdgeConv units and a dense connection. Inspired by the graph convolution network (GCN), [16] proposed a global pooling strategy and a multi-resolution pooling strategy, both of which are based on GCN. These two strategies learn local and global information efficiently.

Moreover, due to the notable performance of the transformer in the computer vision field, some works adopt a transformer mechanism to learn the features of point clouds. The work in [17] proposed a transformer-based architecture (PCT) with offset-attention for point cloud learning. A contemporaneous work [18] proposed another point cloud transformer architecture (PT) that is similar to PointNet++. Both of them perform well on classification and segmentation tasks involving point clouds. Therefore, some subsequent point cloud upsampling works have learned from them to design a backbone. LiUpNet [19] adopted PCT as a backbone to learn point cloud features for LIDAR point cloud upsampling and verified the network’s performance on real-world downstream tasks. KPCONV [20] adopted PT with a dense connection to encode the point clouds for a subsequent feature expansion process. Moreover, some point cloud upsampling works learn point cloud features by modifying the transformer architecture. PUT [3] designed a transformer encoder with Shifted Channel Multi-head Self-Attention to achieve point cloud upsampling efficiently. PU-Edgeformer [4] combined the EdgeConv [15] unit and a transformer as an encoder and adopted a dense connection to extract point cloud features.

## 3. Materials and Methods

**Figure 1:**
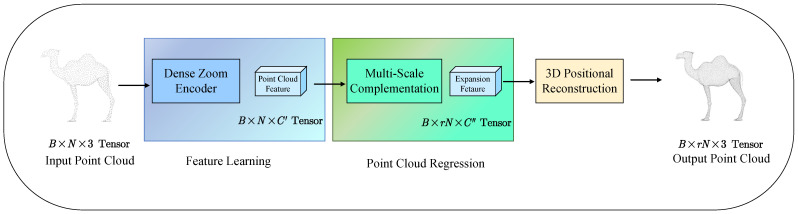
The pipeline of PU-DZMS, which consists of Dense Zoom Encoder and Multi-Scale Complementation.

Figure 1 delineates the comprehensive pipeline of the proposed PU-DZMS network for point cloud upsampling. The architecture operates in two sequential stages to achieve effective feature extraction and density amplification.

The process begins with the input point cloud, represented as a B×N×3 tensor, where B denotes batch size, N is the number of input points, and 3 corresponds to XYZ coordinates, being fed into the Dense Zoom Encoder (DENZE) Module. This first stage module is specifically designed to extract rich, discriminative geometric features directly from the sparse point set. Through its dense connectivity and feature aggregation strategies, the DENZE module generates a refined representation capturing the underlying structure, outputting a point cloud feature tensor of shape $B\times N\times C^{\prime }$.

The second component is the Multi-Scale Complementary Regression (MSCR) Module. Within this module, the acquired point cloud features $B\times N\times C^{\prime }$ first undergo an expansion phase to $B\times N\times C^{\prime \prime }$, increasing the number of potential points by a factor of r effectively. Subsequently, these expanded features undergo multi-scale complementation, where features across different scales interact to capture complementary geometric information and refine the representations.

The multi-scale complemented features are reflected into 3D space through 3D Positional Reconstruction. This step converts the high-dimensional feature vectors $B\times rN\times C^{\prime \prime }$ back into explicit spatial coordinates, yielding the upsampled output point cloud as a B×rN×3 tensor. Thus, PU-DZMS transforms the input sparse point cloud into a densified, more detailed representation through targeted feature learning in the DENZE module followed by point multiplication and precise spatial regression in the MSCR module systematically.

### 3.1. Zoom Encoder

Ensuring that the output point cloud maintains the same distribution as the point cloud relies on capturing both global geometric structures effectively. Although point cloud Transformers are designed to extract global features, their ability to preserve the relationship between global structures weakens when the point cloud becomes sparse, leading to potential misalignment in the reconstructed distribution.

To solve this issue, the Zoom Encoder is designed. Specifically, the input point cloud is disassembled into a fine-grained layer by sampling. The layer contains the detailed information of the point cloud. To enhance the layer feature and the interaction information between points, the point cloud Transformer is employed to encode the layer. The enhanced layer features are then upsampled. Later, the global feature is obtained by combining the recovered layer features.

**Figure 2:**
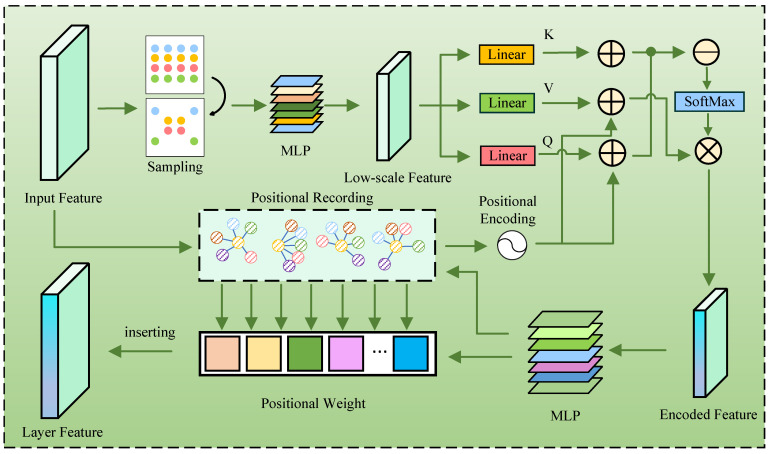
The structure of Zoom-Encoder.

The Zoom Encoder could be seen in Figure 2. The input point cloud is sampled as fine-grained layer by adopting Farthest Point Sampling. Notably, though the layer contains detail information, the layer feature should be enhanced, due to the sparsity of point cloud and the depth of DENZE. Therefore, the layer feature is conveyed to point cloud Transformer block. There are three linear layers transforming the layer feature into key, value and query:

(1) $$\begin{array}{c} Q\;\;=\;\;Linear\left(P_{i}\right)\\ K\;\;=\;\;Linear\left(P_{i}\right)\\ V\;\;=\;\;Linear\left(P_{i}\right) \end{array}$$

where Linear() denotes the linear operation and $P_{i}$ represents the fine-grained feature after Farthest Point Sampling. It is worth noting that the layer contain details information, which should be aggregated to renew the key and value by filtration mechanism.

The positional encoding feature is obtained from the original point cloud. Referring to vector attention [18], the layer feature is enhanced by:

(2) $$F_{i}=Softmax\left(\frac{P+Q-K_{e}}{\sqrt{d_{k}}}\right)\left(P+V_{e}\right)$$

*P* denotes the positional encoding feature, and $K_{e}$ and $V_{e}$ denote the renewed key and value, respectively. $d_{k}$ denotes the dimension of $K_{e}$. To obtain global feature, it is necessary that the tensor of each layer of enhanced feature should be recovered to the same size as original point cloud. For this requirement, the sampling points of the last layer are grouped by inquiring their positions in the original point clouds, and the mathematical expectation of the distances between the centroid and the group members is adopted as weight. Therefore, the virtual points with neighbor information could be obtained by:

(3) $$p_{l}=\sum_{t=1}^{T}F_{i}^{l-1}w_{t}$$

where $F_{i}^{l-1}$ denotes the last layer feature, and $w_{t}$ denotes the weight of the *t*-th group member. The tensor scale of layer feature is recovered by adding the virtual points.

### 3.2. Dense Zoom Encoder

**Figure 3:**
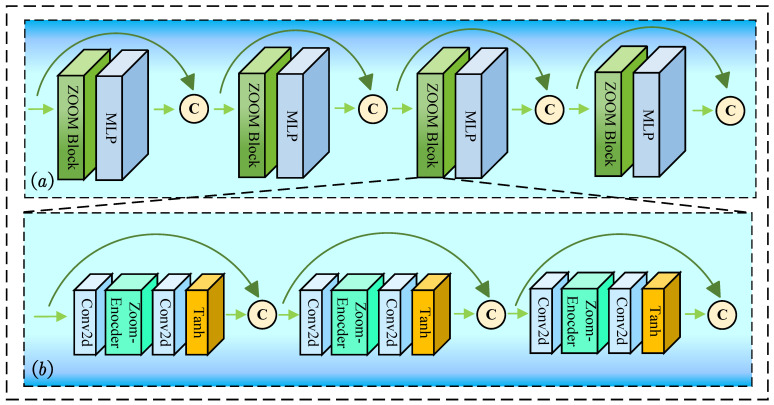
The structure of Dense Zoom Encoder in (**a**) and the structure of ZOOM Block in (**b**).

Figure 3a shows the architecture of DENZE and Figure 3b shows the architecture of ZOOM Block. The Dense Zoom Encoder consists of four residual connection units, which contains Zoom Blocks and MLPs. The ZOOM Block employs layered convolutions and nonlinear transformations to extract local geometric features. The subsequent MLP nonlinearly projects these features into a higher-dimensional latent space, enhancing representational separability. Moreover, inspired by EdgeConv [15], the output tensor of each stage is concatenated with the input tensor to maintain the reliability of the features.

These operators perform channel-wise concatenation, enabling persistent combination of structural details from shallow layers with semantic features, which come from deeper layers. The unidirectional dataflow propagates features from input to output without recurrence strictly, forming a rich feature optimized for multi-scale geometry encoding.

The ZOOM Block constitutes the fundamental processing unit of the DENZE, being responsible to feature learning in point cloud upsampling tasks. The unit sequentially integrates a two-dimensional convolution layer for spatial correlation extraction, an Encoder module for nonlinear feature embedding, followed by another Conv2d layer to refine spatial relationships, and culminates with a Tanh activation function. This process could be described as:

(4) $$F_{1}=\;ZE\left(Conv2d\left(F_{0}\right)\right)$$

(5) $$F_{2}=Tanh\left(Conv2d\left(F_{1}\right)\right)$$

where Conv2d() denotes the 2D convolution layer, ZE() denotes the Zoom-Encoder, Tanh() denotes the tanh activation function. The Tanh operation enhances gradient stability while bounding activation ranges, mitigating vanishing gradient risks during deep propagation. There are three units in the ZOOM Block; the outputs of each unit are fused together by residual connection. These operations enhance local geometric sensitivity while introducing multi-resolution features for zoom-based point cloud enhancement.

### 3.3. Multi-Scale Complementary Regression

**Figure 4:**
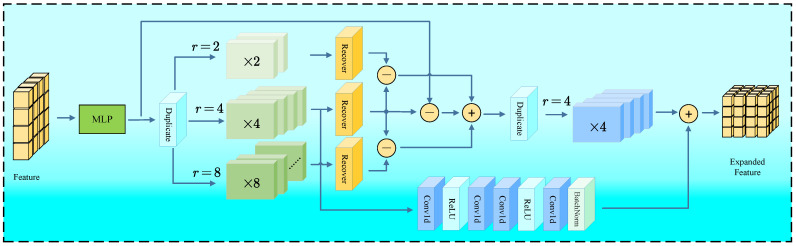
The structure of MSCR module. MSCR module combines different scale feature to reduce the sparse regions in the generated point cloud.

The Multi-Scale Complementary Regression module is designed to expand the point-wise features generated by DENZE, and regress the dense point cloud. The details of the MSCR module are shown in Figure 4. The input feature is first transformed through an MLP. Then the processed feature is duplicated across three parallel branches with distinct geometric scaling objectives, specifically targeting scaling factors $r\in \left\{2,4,8\right\}$. This process could be described as:

(6) $$F_{r}=Dup\left(MLP\left(F_{ZE}\right),r\right)\;\forall r\in \left\{2,4,8\right\}$$

where Dup() denotes the Duplicate operator, and $F_{ZE}$ denotes the feature coming from DENZE.

In each scaling branch, the Recover Module executes sequential processing. This module incorporates a self-attention mechanism that enhances feature significance based on contextual relationships within the point cloud dynamically, followed by convolutional operations designed to reorganize spatial relationships according to each target scale. This combined strategy adapts features to their target scale while preserving structural coherence. This process could be described as:

(7) $$F_{r}^{\prime }=Recover\left(F_{r},4\right)\;\forall r\in \left\{2,4,8\right\}$$

where Recover() denotes the Recovery operator. The architecture then engages its core complementation mechanism, which is achieved by cross-scale residual connection. The features from adjacent scaling branches pass through differential comparison through subtractive operations, explicitly capturing scale-specific geometric variances:

(8) $$\Delta F_{0-4}=F_{4}^{\prime }-MLP\left(F_{ZE}\right)$$

(9) $$\Delta F_{2-4}=F_{4}^{\prime }-F_{2}^{\prime }$$

(10) $$\Delta F_{8-4}=F_{4}^{\prime }-F_{8}^{\prime }$$

These differential outputs are integrated through summation, generating a consolidated feature representation that synthesizes complementary geometric information across multiple scales:

(11) $$F_{com}=\Delta F_{2-4}+\Delta F_{8-4}+\Delta F_{0-4}$$

This aggregated feature tensor is subsequently expanded with scale r=4. The four-times duplicated feature, which is generated in the first duplication operator, flows through the residual connection with convolutional processing. Moreover, the connection contains convolution layers, batch normalization and nonlinear activations, and it refines geometric representations progressively. This process could be described as:

(12) $$F_{\exp}=Dup\left(F_{com},4\right)+Res\left(F_{4}\right)$$

where Res() denotes the residual connection.

The expand feature is conveyed to 3D Positional Reconstruction to obtain the point cloud under the Cartesian coordinate. Three-dimensional Positional Reconstruction contains several MLP layers, yielding the upsampled point cloud that exhibits enhanced spatial distribution faithful to the underlying surface geometry.

### 3.4. Loss Function

The loss function of PU-DZMS consists of two parts. The first part is Chamfer Distance (CD), which represents the minimum distance summation of the points in one set to the other set:

(13) $$\begin{array}{c} L_{CD}=\frac{1}{N_{1}}\sum_{p\in S_{1}}\min_{q\in S_{2}}\left({∥p-q∥}_{2}^{2}\right)+\frac{1}{N_{2}}\sum_{q\in S_{2}}\min_{p\in S_{1}}\left({∥q-p∥}_{2}^{2}\right) \end{array}$$

where $S_{1}$, $S_{2}$ denote the output point cloud and groundtruth (GT) point cloud, respectively. *p*, *q* denote the point in the output point cloud and GT, respectively, and $N_{1}$, $N_{2}$ denote the number of points in the output point cloud and GT, respectively.

The second part is a parameter regularization term that can prevent model overfitting. Therefore, the loss function of PU-DZMS could be described as follows:

(14) $$L=\alpha L_{CD}+\beta {∥\theta ∥}_{2}$$

where θ denotes the parameters of the model. α and β denote the weight factors of the loss items.

## 4. Results

### 4.1. Dataset and Metrics

To verify the performance of our proposal, the PU-GAN dataset and PU-Net dataset are adopted to train and test PU-DZMS.

The PU-GAN dataset leverages 147 synthetic mesh models from the Visionair repository, partitioned into 120 training and 27 validation samples representing diverse surfaces—including smooth organic shapes and sharp-edged mechanical structures—each uniformly sampled to create input/ground-truth pairs via Poisson disk sampling targeting 4× and 16× upsampling ratios.

The PU-Net dataset is curated from 60 distinct 3D mesh models sourced from the Visionair repository. This collection encompasses diverse geometric typologies ranging from smooth non-rigid surfaces including the Bunny model to steep rigid structures such as the Chair model. The dataset was partitioned into 40 models for training and 20 for testing. Each training model contributed 100 surface patches, resulting in a comprehensive training corpus of 4000 patches.

Following [7], the metrics chosen for evaluation are Chamfer Distance (CD) [21], Hausdorff Distance (HD) [21] and Jensen–Shannon Divergence (JSD) [22]. CD measures the similarity between two point cloud sets by computing the average nearest-neighbor distance from one set to the other, while HD finds the maximum nearest-neighbor distance between two point cloud sets, capturing the worst-case discrepancy. Different from the former two metrics, JSD quantifies the similarity between two probability distributions based on their KLD values.

### 4.2. Implementation Details

PU-DZMS is trained with a batch size of 24 and a batch size of 64 for 100 epochs on the PU-GAN dataset and PU-Net dataset, respectively. The Adam optimizer is utilized with an initial learning rate of 0.001, which reduces gradually by following the step schedule with step size s=30 and decay factor θ=0.1 for the PU-Net dataset, with milestones m=[50, 80] and decay factor θ=0.2 for the PU-GAN dataset. Moreover, the scale of upsampling is set to 4, from 256 to 1024 points for the PU-GAN dataset and from 1024 to 4096 points for the PU-Net dataset. For the loss function, the weights of loss items are set to α,β=1.0, 0.1, respectively. All of the training works are conducted on a single NVIDIA RTX 3090 GPU.

### 4.3. Quantitative and Qualitative Result Analysis

We choose PU-Net [1], MPU [2], PU-GAN [6], PU-GCN [5], Dis-PU [9] and PU-Transformer [3] as SOTA methods. The upsampling scale factor is set to 4, from 256 points to 1024 points. The quantitative result is shown in Table 1. Our method achieves strong reconstruction fidelity with a leading CD of 0.227, outperforming Grad-PU’s 0.248 and PU-Transformer’s 0.276. We similarly attain the best HD of 2.519, demonstrating good local geometric stability compared to alternatives. While RepKPU achieves a marginally better JSD of 0.039, our result remains competitive and exceeds baseline methods like PU-GAN on the whole, confirming a high preservation of spatial distribution characteristics. This performance advantage stems from two key mechanisms. One is that Explicit geometric feature learning suppresses boundary distortion; the other is that Multi-scale feature integration ensures geometric consistency across hierarchical levels. Regarding model efficiency, our model yields moderate parameters, as 79.5 KB versus PU-GCN’s minimum 76.4 KB, but higher memory with 17.1 MB versus PU-GCN’s 1.9 MB. Moreover, our approach delivers a 12.5% improvement in CD and 7.7% improvement in HD over the lightweight PU-GCN benchmark.

PU-DZMS is also verified on the PU-Net dataset, and the experiment result is presented in Table 2. Our approach demonstrates competitive reconstruction quality, achieving a CD metric of 0.308, ahead of conventional methods like PU-GAN, PU-GCN, and Dis-PU, although PU-Transformer achieved the optimal CD of 0.276. Similarly, our method attained an HD of 4.175, outperforming earlier techniques such as MPU and PU-Net, while PU-Transformer leads with 3.019. For distribution fidelity measured by JSD, our result ranks as the third best, surpassing benchmarks including PU-GAN and PU-Net, with PU-Transformer and Dis-PU securing superior scores. This result demonstrates that our method maintains this strong performance while generalizing effectively across diverse test scenarios, outperforming other traditional methods in geometric fidelity and generalization capability on the PU-Net benchmark.

However, PU-DZMS consumes 17.1MB memory, exceeding PU-GCN. This hinders deployment on edge devices, a failure scenario for real-time applications.

**Figure 5:**
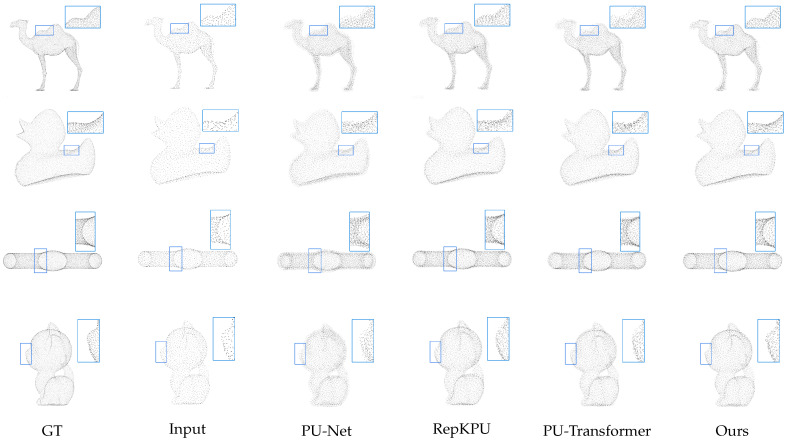
Qualitative results on PU-GAN dataset. We remark some local details with blue rectangles to highlight the performance of our approach.

Figure 5 presents qualitative comparisons on the PU-GAN dataset, demonstrating the reconstruction capabilities of our approach against representative methods including PU-Net, RepKPU, and PU-Transformer. Sparse input point clouds lack fine details, yet all upsampled results show denser outputs. PU-Net generates notably uneven surfaces with visible clustering artifacts, particularly evident within the highlighted regions of the camel’s leg joints where points fail to capture natural bone articulation. RepKPU produces smoother but overly regularized surfaces, attenuating sharp features like the duck’s wing ridges, the camel’s back, and the kitten’s nose. PU-Transformer maintains stronger global shape consistency but struggles with precise curvature reproduction, distorting subtle features such as the capsule’s terminal symmetry.

For the camel leg joints in Figure 5, PU-DZMS generates uneven point distributions compared to ground truth, though it outperforms PU-Net and RepKPU. The joints represent non-smooth manifolds with abrupt curvature changes. The current Zoom Encoder prioritizes global consistency, potentially underweighting high-frequency local deformations.

### 4.4. Robustness Testing

Following robustness evaluation in [23], where Gaussian noise corruptions from 0.5% to 2.0% were introduced to the PU-GAN dataset. The result could be seen in the Table 3. Our method demonstrates good robustness against point cloud perturbation. At the mildest 0.5% noise level, our approach achieves the best CD of 0.273 and optimal JSD of 6.307 while maintaining highly competitive HD at 4.489, surpassed only marginally by PU-Transformer’s 4.025 in this single metric. Under moderate 1.0% noise intensity, our solution leads all benchmarks with a CD of 0.322, exceeding the second-ranked Dis-PU by 50.3%; it also achieves a leading HD of 5.578, outperforming PU-Transformer by 6.1%, and the best JSD of 8.198. This trend continues at 1.5% noise where we maintain SOTA performance across all metrics: CD 0.658, HD 8.593, and JSD 10.167. Even under severe 2.0% noise corruption where Dis-PU slightly edges ahead in CD by 0.044, our approach shows better geometric stability with an HD of 9.924, compared to Dis-PU’s 11.582 and better JSD, 17.344 versus 19.735 on the whole. Critically, our architecture exhibits the slightest performance degradation when noise escalates, with average metric deterioration rates of merely 0.614 for CD, 5.435 for HD, and 1.104 for JSD, which are all lower than any method. This comprehensive validation confirms our model’s ability to preserve structural integrity under noise perturbations well.

**Figure 6:**
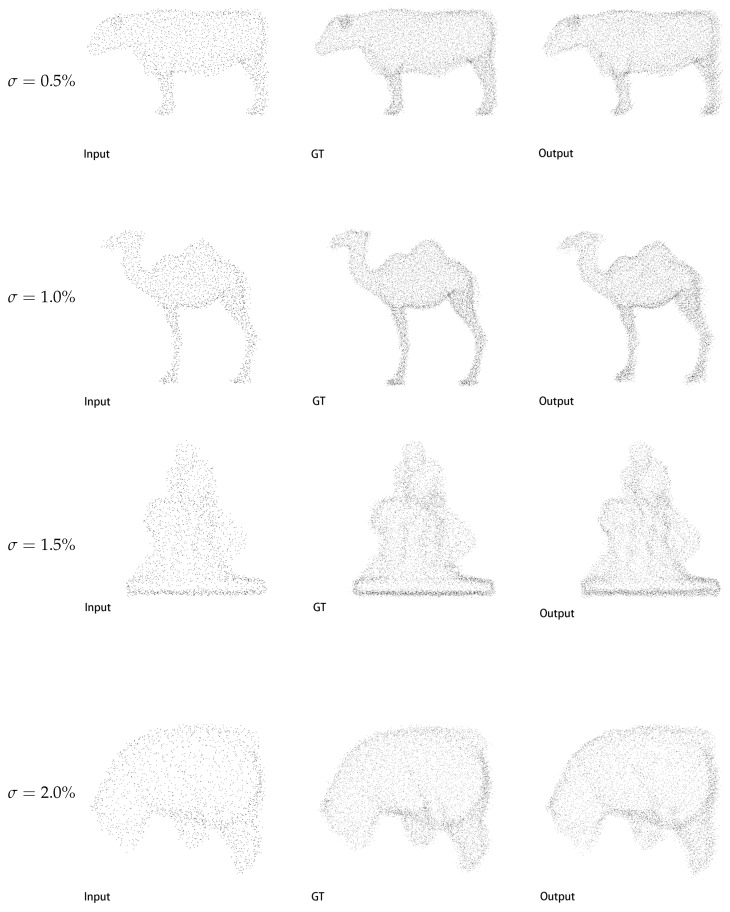
Qualitative results on PU-GAN dataset about the robustness.

Figure 6 shows our method’s robustness under the Gaussian noise with different variance from 0.5% to 2.0%. Input quality degrades progressively, exhibiting increasing point scattering as σ rises. Nevertheless, our outputs maintain coherent geometric topology with sharp boundary definition across all noise levels consistently, approximating ground truth integrity even under severe σ=2.0% corruption, where inputs approach structural dissolution. Moreover, our reconstructions preserve fine-scale structural features lost in noise-corrupted inputs. This persistent retention of original shape characteristics is achieved by obviously suppressing outlier points and sustaining contour precision under the noise. The experiment result confirms that our DENZE feature learning and MSCR module suppress the noise while preserving intrinsic geometric priors.

### 4.5. Evaluation on the KITTI Dataset

**Figure 7:**
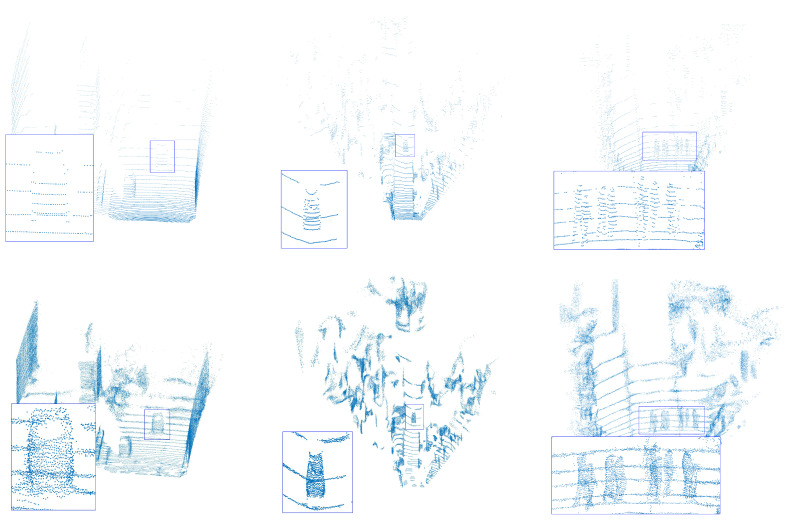
The visualization results on the KITTI dataset. The first row is the input point cloud, and the second row is the dense point cloud generated by PU-DZMS.

Figure 7 shows the evaluation results on the KITTI dataset. The instances are marked with blue rectangles. It could be seen that our PU-DZMS could generate a dense point cloud for the sparse regions of instances, such as the cars in the first and second scenes. For the pedestrian in the third scene, the points locate following the natural outline of instances. These results show the generalization ability of our PU-DZMS for the real-world point cloud.

### 4.6. Ablation Study

#### 4.6.1. Evaluation on the Designed Modules

The ablation study result is recorded in Table 4. The baseline model with dense convolution and single-scale regression achieves CD 0.2648, HD 6.876, and JSD 0.0498. Replacing single-scale with multi-scale regression while retaining dense convolution degrades performance, increasing CD to 0.2898 despite a minor HD reduction to 5.861. This indicates Multi-Scale Regression with complementary feature learning mechanisms could enhance reconstruction.

Integrating Dense Zoom Encoder feature learning with single-scale regression delivers clear gains; CD drops to 0.2401, HD reduces to 2.9530, which is 57% lower than baseline, and JSD improves to 0.0408. These improvements suggest that DENZE offers advantages in geometric representation over conventional convolutions. The full model, combining DENZE and Multi-Scale Regression, achieves the best performance in this study, with a CD of 0.2266, an HD of 2.5130, and a JSD of 0.0396.

This result demonstrates that DENZE and MSCR modules play an important role in point cloud upsampling. First, DENZE contributes primary performance gains by learning rich geometric feature. Second, MSCR provides conditional advantages; it combines the geometric details of the dense point cloud in different zoom scales, which could reduce the blank spaces. Therefore, both components are necessary for optimal point cloud reconstruction.

#### 4.6.2. Evaluation on the Hyper-Parameters

The ablation study in Table 5 demonstrates the importance of the depth of DENZE and comprehensive scale of the MSCR module for optimal point cloud reconstruction. When maintaining the full multi-scale combination [2, 4, 8], progressively increasing Dense Zoom Block layers from two to three improves reconstruction fidelity, evidenced by CD reduction from 0.2551 to 0.234, HD reduction from 5.438 to 3.213, and JSD improvement from 0.048 to 0.042. Further increasing to the four-layer configuration achieves the best performance observed in our experiments, with CD dropping to 0.2266 and HD plunging to 2.519, establishing new state-of-the-art benchmarks across all evaluation metrics. This progressive refinement confirms that deeper hierarchical processing enhances the network’s capacity to model complex geometric relationships, enabling increasingly precise reconstruction of structural details through advanced feature extraction and representation learning capabilities.

Under the optimal four-layer architecture, the selection of scale groups proves equally decisive for reconstruction accuracy. Utilizing only single-scale representation with scale [4] yields compromised performance at CD 0.240 and HD 2.953 on the whole. Incomplete scale combinations like [2, 4] demonstrate CD 0.251, while discontinuous groupings such as [4, 6] and [4, 8] produce similar performance ceilings at CD 0.249 and CD 0.248, respectively. Near-optimal results emerge with the continuous but incomplete sequence [2, 4, 6] achieving CD 0.230 and HD 2.603. Moreover, only the complete hierarchical scaling [2, 4, 8] configuration delivers peak performance with CD 0.227 and HD 2.513, outperforming the [2, 4, 6] configuration by 1.4% in CD and 3.2% in HD while simultaneously achieving the best JSD of 0.0396.

#### 4.6.3. The Analysis of Module-Level Computational Complexity

Table 6 shows the evaluation on the computational complexity of designed modules. The input size of tensor is 1 × 256 × 3. It could be seen that DENZE has relatively high computational complexity, due to the dense connection and Transformer mechanism. MSCR has computational complexity with GFOLPs of 29.7 and latency of 12.1 ms, which are approximate to DENZE. Therefore, the high computational complexity is a challenge that PU-DZMS should face in future work.

## 5. Conclusions

PU-DZMS achieves point cloud upsampling through its dual-module synergy. The DENZE adopts hierarchical feature stacking via progressively deepened Zoom Blocks, each integrating residual connections and localized convolution operators. This architecture preserves geometric details by establishing a cross-layer feature propagation method, where shallow structural details persistently inform deeper semantic representations. Moreover, the MSCR module implements different scale progressions as its operational paradigm. By referring differential feature interactions across scaling pathways and integrating them through learned residual mappings, MSCR captures complementary geometric contexts that single-scale frameworks could not represent. The intrinsic hierarchical processing inherent in this dual-module design further enables robust noise suppression, where multi-stage feature refinement isolates structural signals from perturbations progressively.

However, PU-DZMS consumes a considerable amount of memory, due to the dense connection and multi-scale middle variate. Therefore, our future work focuses on designing a point cloud upsampling network with light weights.

## Figures and Tables

**Table 1:** Quantitative results on PU-GAN dataset. The best results are highlighted with boldface, and the downarrows mean small value is optimal.

Methods	CD↓ ($10^{-3}$)	HD↓ ($10^{-3}$)	JSD↓	Param.↓ (KB)	Model↓ (MB)
PU-Net	0.836	7.178	0.079	814.3	10.1
MPU	0.621	6.755	0.066	77.8	92.5
PU-GAN	0.514	5.282	0.057	516.0	9.8
PU-GCN	0.354	5.173	0.051	**76.4**	**1.9**
Dis-PU	0.293	4.003	0.043	1047.5	13.2
PU-Transformer	0.276	3.019	0.043	969.9	18.4
Grad-PU	0.248	2.618	0.042	67.1	7.2
RepKPU	0.248	2.876	0.039	989.5	10.11
PUDM	**0.218**	2.546	0.047	28.65	12.84
Ours	0.227	**2.513**	**0.040**	79.5	17.1

**Table 2:** Quantitative results on PU-Net dataset. The best results are highlighted with boldface, and the downarrows mean small value is optimal.

Methods	CD↓ ($10^{-3}$)	HD↓ ($10^{-3}$)	JSD↓	Param.↓ (KB)	Model↓ (MB)
PU-Net	1.175	16.782	0.199	814.3	10.1
MPU	0.937	12.211	0.117	77.8	92.5
PU-GAN	0.708	9.532	0.084	516.0	9.8
PU-GCN	0.574	7.569	0.073	**76.4**	**1.9**
Dis-PU	0.496	6.268	0.071	1047.5	13.2
PU-Transformer	**0.276**	3.019	0.064	969.9	18.4
PUDM	0.280	3.458	0.047	28.65	12.84
Ours	0.308	**4.175**	**0.043**	79.5	17.1

**Table 3:** Robustness testing results with 0.5%, 1.0%, 1.5% and 2.0% noisy level on PU-GAN dataset. The best results are highlighted with boldface, and the downarrows mean small value is optimal.

Methods	σ= 0.5%			σ= 1.0%			σ= 1.5%			σ= 2.0%		
	CD↓ (10^−3^)	HD↓ (10^−3^)	JSD↓ (10^−2^)	CD↓ (10^−3^)	HD↓ (10^−3^)	JSD↓ (10^−2^)	CD↓ (10^−3^)	HD↓ (10^−3^)	JSD↓ (10^−2^)	CD↓ (10^−3^)	HD↓ (10^−3^)	JSD↓ (10^−2^)
PU-Net	1.443	13.329	9.134	1.082	15.100	13.467	1.238	16.337	22.235	1.505	19.895	34.199
PU-GAN	0.358	5.551	7.351	0.447	10.383	9.296	0.750	13.265	17.082	1.188	18.631	23.827
PU-GCN	0.597	8.271	8.663	0.762	9.687	15.740	0.943	10.705	13.532	1.219	13.971	21.236
Dis-PU	0.492	6.033	7.323	0.646	8.099	8.487	0.864	10.541	12.816	**0.843**	11.582	19.735
PU-Transformer	0.480	**4.025**	8.241	0.682	5.939	9.622	0.743	9.894	13.173	1.077	10.256	20.444
Ours	**0.273**	4.489	**6.307**	**0.322**	**5.578**	**8.198**	**0.658**	**8.593**	**10.167**	0.887	**9.924**	**17.344**

**Table 4:** Ablation study result about the performance of DENZE and MSCR module. The best results are highlighted with boldface, and the downarrows mean small value is optimal.

Model	Feature Learning	Point Cloud Regression	CD↓ ($10^{-3}$)	HD↓ ($10^{-3}$)	JSD ↓
Model1	Dense Convolution	Single Scale	0.265	6.876	0.050
Model2	Dense Convolution	Multi-Scale	0.290	5.861	0.050
Model3	Dense Zoom Encoder	Single Scale	0.240	2.953	0.041
Ours	Dense Zoom Encoder	Multi-Scale	**0.227**	**2.513**	**0.040**

**Table 5:** Ablation study result on the layers of ZOOM Block in DENZE and the scale groups of MSCR module. The best results are highlighted with boldface, and the downarrows mean small value is optimal.

Model	Layer Groups	Scale Groups	CD↓ ($10^{-3}$)	HD↓ ($10^{-3}$)	JSD ↓
Model1	2 Layers	[2, 4, 8]	0.255	5.438	0.048
Model2	3 Layers	[2, 4, 8]	0.234	3.213	0.042
Model3	6 Layers	[2, 4, 8]	0.227	2.524	**0.039**
Model4	8 Layers	[2, 4, 8]	0.232	2.518	0.040
Ours	4 Layers	[2, 4, 8]	**0.227**	**2.513**	0.040
Model5	4 Layers	[4]	0.240	2.953	0.041
Model6	4 Layers	[2, 4]	0.251	3.098	0.042
Model7	4 Layers	[4, 6]	0.249	2.952	0.048
Model8	4 Layers	[4, 8]	0.248	2.814	0.046
Model9	4 Layers	[2, 4, 6]	0.230	2.603	0.041
Ours	4 Layers	[2, 4, 8]	**0.227**	**2.513**	**0.040**

**Table 6:** The module-level computational complexity analysis.

Module	GFLOPs	Latency (ms)
DENZE	38.2	15.4
MSCR	29.7	12.1
3D Reconstruction	3.8	1.5
Total	71.7	29.0

## Data Availability

The data used to support the findings of this study are available from the corresponding author upon request.
